# Very High Cycle Fatigue (VHCF) Characteristics of Carbon Fiber Reinforced Plastics (CFRP) under Ultrasonic Loading

**DOI:** 10.3390/ma13040908

**Published:** 2020-02-18

**Authors:** Wenbin Cui, Xuan Chen, Chao Chen, Li Cheng, Junliang Ding, Hui Zhang

**Affiliations:** Aeronautics Engineering College, Air Force Engineering University, Xi’an 710038, China; yy2437704026@163.com (W.C.); chen1264692561@162.com (C.C.); cheng_qiaochu@foxmail.com (L.C.); 18700457532@163.com (J.D.); rxwyxz@163.com (H.Z.)

**Keywords:** carbon fiber reinforced plastics, very high cycle fatigue, S-N curve, damage morphology, evolution threshold value

## Abstract

A liquid nitrogen cooling system was developed to ensure the successful ultrasonic testing of composite materials to characterize the very High Cycle Fatigue (VHCF) of carbon fiber reinforced plastics (CFRP). The fatigue failure of CFRP occurs even in the very high cycle range and there is no traditional fatigue limit. The S–N curve of the CFRP presents a step whose characteristics appear in the transition between high cycle and very high cycle fatigue. The damage evolution of CFRP in the same field of view is investigated. The morphology of damaged CFRP composites under ultrasonic loading is described by three characteristics: matrix damage at the intersection of fiber bundles, near fiber bundle parallel section matrix cavity and matrix penetration. With the increasing of cycles, the damage process is also presented in turn according to these three characteristics. The post-fatigue bending modulus changed significantly from the pre-fatigue values, indicating that the VHCF had a considerable impact on the mechanical properties of the composite. An evolution threshold was introduced from the S–N curve to determine the fatigue evolution law from the high cycle regime to the very high cycle regime.

## 1. Introduction

Carbon fiber reinforced plastics (CFRP) are widely used in the aerospace field due to their advantages, such as a high specific strength and modulus, a good fatigue resistance, a low friction coefficient and wear rate, and good thermal and electrical properties. However, the high fabrication cost and the very high maneuverability of modern aircraft easily drives the number of fatigue cycles of composite parts above 10^7^ during their service life and very high cycle fatigue (10^7^–10^11^) must be considered [1,2,3,4,5,6,7]. The fatigue has been extensively studied worldwide. However, most of the current research on composite materials focuses on the static failure or the low cycle fatigue, whereas experimental research on high cycle fatigue (HCF) and very high cycle fatigue (VHCF) is still very scarce [8]. The testing frequency [9] and the heating of the specimen [10] are key factors restricting the fatigue study of composite materials. Carlile et al. [11] and Curtis et al. [12] considered these issues to reduce the fatigue strength of APC-2/AS4 laminates. In 1997, Couillard et al. [13] studied the fatigue performance of CFRP under low frequency loading. In 2006, Silvain et al. [14] studied carbon fiber composite materials at low frequencies within 0.5–10 Hz and high frequencies within 57–158 Hz and extended the loading cycles to over 10^9^. They concluded that damage still occurs in the VHCF regime. In 2013, Gude et al. [15] studied the failure mechanism of carbon fiber reinforced plastics in the VHCF regime. Their results showed that the specimens break perpendicularly to the ply direction in the VHCF regime and cause a significant decrease in stiffness. In 2014, Adam et al. [9] completed a very high cycle fatigue study of [90/0] s glass fiber braided composites with cyclic four point bending at frequencies within 50–80 Hz. Their results indicated that the crack propagation along the direction of the thickness was retarded under a low load and that delamination was delayed. In 2015, Backe et al. [16] carried out a very high cycle fatigue study on CFRP composites using an ultrasonic fatigue test method at a frequency of 20 kHz and pointed out that CFRP still experience fatigue failure under low amplitude loads and a long duration. The fatigue on aircraft is mainly caused by forced and non-synchronous vibration due to various aerodynamic and mechanical reasons. The stress amplitude is relatively low, but the frequency is high. This leads to a large number of cycles accumulated over a short amount of time, which causes a major hidden threat to aviation equipment safety. At present, the very high cycle fatigue of metal materials has been well studied, but research on the very high cycle fatigue of composite materials needs to be accelerated.

In the present work, we developed a liquid nitrogen cooling system to ensure smooth ultrasonic loading. The CFRP specimens were subjected to an ultrasonic three point bending fatigue test and the S–N curves corresponding to the high cycle and the very high cycle fatigue were established. Moreover, the microstructure and the failure morphology of the specimens were examined. The characteristics and the laws of evolution of fatigue damage were determined in combination with the low cycle fatigue. Static three point bending tests were performed on the specimens before and after the test and the influence of the VHCF on the flexural modulus of the material was determined. In addition, the strain release energy G was obtained from the S–N curves, and a threshold value of HCF to VHCF evolution was established.

## 2. Materials and Methods

### 2.1. CFRP Specimens

We used a carbon fiber/epoxy resin composite material (carbon fiber: T300, layering order: [90°/0] s, fiber volume fraction: 54%, porosity: 3%) produced via hot-press molding. The relevant properties are listed in Table 1.

**Figure 1:**
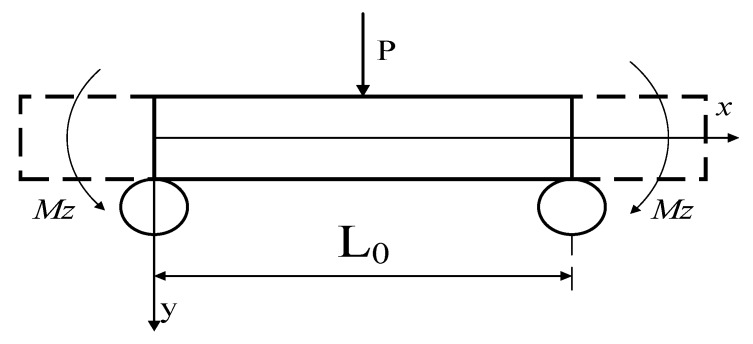
Loading of the carbon fiber reinforced plastics (CFRP) specimen.

**Figure 2:**
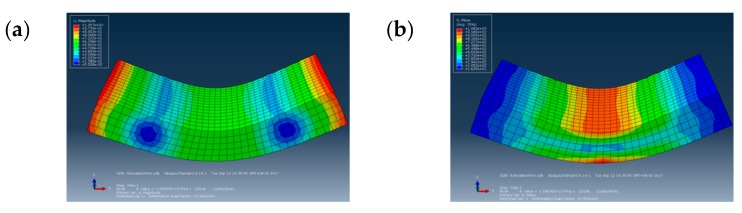
(**a**) Displacement and (**b**) stress simulation results for the CFRP specimens.

The force analysis of the CFRP specimen of the three point bending tests is shown in Figure 1. The ABAQUS 6.14–1 CAE software (Dassault Simulia Inc, Providence, USA) was used for the modeling and the simulation of the CFRP specimens to determine a size suitable for ultrasonic loading, as shown in Figure 2.

The analysis showed that a specimen size of 29 × 14 × 4 mm and a distance of L_0_ = 16 mm between the two pivots yields a resonance frequency of 20106 Hz, which meets the requirements of a very high cycle test. According to the anisotropic natural frequency equation [17], we obtained the size of the simulated test piece:

(1) $$Y^{⁗}+b^{2}(r^{2}-s^{2})Y^{\prime \prime }-b^{4}r^{2}a^{2}Y=0.$$

(2) $$\phi^{⁗}+\frac{NS_{11}L_{0}}{I_{Z}}Y^{\prime \prime \prime }+\frac{S_{11}\rho A\omega ²L_{0}³}{I_{Z}}Y^{\prime }=0.$$

among them, $r²=I_{z}S_{66}\rho \omega ²$; $r²=I_{z}S_{66}\rho \omega ²$; $S²=S_{11}kN$; $a²=\frac{S_{11}}{S_{66}}Ak²$. *r*, *S*, *a*: letters used to facilitate the calculation. A: Test specimen cross-sectional area. ω: Specimen natural frequency. k: Shear correction factor, this article uses 7/9.

Using the boundary conditions, the characteristic equation becomes:

(3) sin(bβ)=0.

(4) $$\beta =\frac{1}{\sqrt{2}}{\left\{{\left[(r²-s²)²+4m²r²\right]}^{\frac{1}{2}}+(r²-s²)\right\}}^{\frac{1}{2}}.$$

(5) $$b^{2}\beta^{2}={\left(n\pi \right)}^{2}.$$

*n* is the natural frequency order.

**Figure 3:**
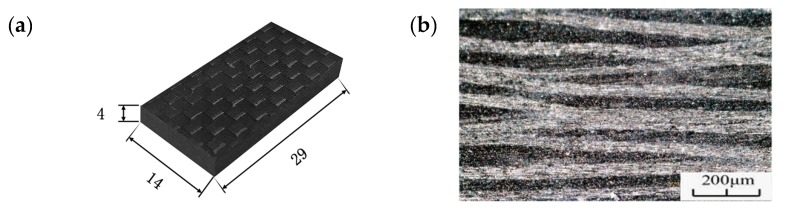
(**a**) Dimensions and (**b**) microscope image of the CFRP specimen.

A Matlab 2014 program combining Equations (1)–(5) was created for the analysis of the simulation. When n = 4 to determine the fourth order resonance frequency ω = 20116 Hz of the test piece, the size of the test piece was reasonable and could be used in a VHCF test, as shown in Figure 3a. No manufacturing holes and no delamination was present, according to the observations with an optical microscope. The microscope image of the CFRP specimen is shown in Figure 3b.

### 2.2. Test Apparatus

**Figure 4:**
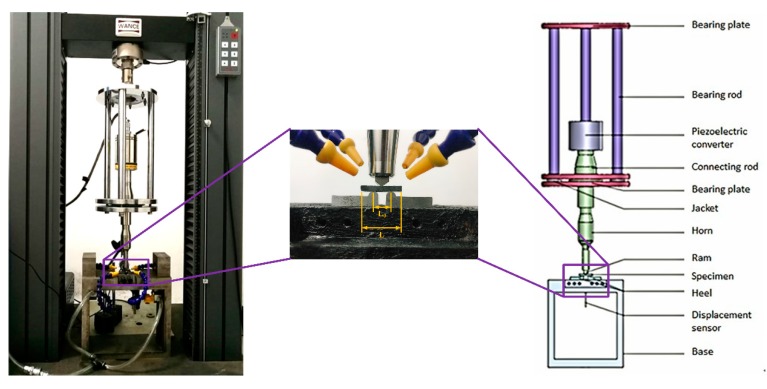
Ultrasonic three point bending fatigue test device.

High cycle fatigue tests on composite materials are mostly performed using servo hydraulic testing devices at frequencies within 3–10 Hz. However, even at a frequency of 10 Hz, it can take up to 116 days to complete 10^8^ cycles without interrupting the experiment, which is too expensive and too time consuming [18]. In this work, we independently developed an ultrasonic three point bending test device to carry out the very high cycle fatigue test of the CFRP specimens, as shown in Figure 4. The test device includes a piezoelectric converter, a connecting rod, a luffing rod, an indenter, a load bearing device, a base, and a measurement and control system (HC SONIC, Hangzhou, China) [19,20,21].

The ultrasonic three point bending fatigue test device is based on the principle of resonance to (i) ensure that the specimen and the test system have the same resonance frequency and (ii) realize the composite loading of different static and dynamic loads. A numerical control ultrasonic generator (HC SONIC, Hangzhou, China) converts a sinusoidal electrical signal at 50 Hz into an ultrasonic signal at 20 kHz. After the transformation performed by the converter, the signal can be converted into a mechanical vibration at the same frequency, and then the vibration amplitude of the test can be amplified by a horn. The converter, the connector, the rod, and the ram constitute the longitudinal resonance system. The longitudinal vibration load is then transferred to the specimen through the ram and so that the specimen experiences bending vibration.

At the beginning of the test, a microcomputer controlled electronic universal testing machine was used to apply a static load to the test piece through the indenter. During the test, a MTI-2100 optical fiber displacement sensor was used to measure the bottom displacement of the test piece. The frequency response was recorded up to 500 kHz with an accuracy of 0.1 μm and a peak to peak range. In normal tests, the resonance frequency was 20 ± 0.5 kHz, which causes the bending resonance of the specimen while the displacement remains unchanged. When the frequency drops sharply below 19.5 kHz and the displacement changes significantly, the system automatically interrupts the test. At this time, it can be determined whether or not the test piece is damaged. The computer control system records the stress amplitude during the test and the cycle time of the specimen when the fracture occurs. During the test, a portable microscope (50×–1000×, BIJIA, Guangzhou, China) was used to track the longitudinal section of the CFRP specimen and perform in situ observations. A liquid nitrogen cooling system is used to cool the specimen surface, and a FLIR T630 infrared camera is used to obtain real time temperature measurements of the CFRP specimen. The temperature field data around the test piece is monitored and collected to ensure that the test piece temperature is always in the glass domain during ultrasonic loading. Below the state transition temperature, the sampling frequency is 30 Hz to ensure a normal evolution of the test.

### 2.3. Cooling Method

Since the ultrasonic loading test uses a vibration frequency of 20 kHz, the high frequency vibration promotes the friction between the inner layers of the specimen, which is most intuitively reflected in the increase in the specimen temperature. The composite material is more affected by temperature due to its lower thermal conductivity. Hosoi et al. [22,23,24] studied the high cycle fatigue of quasi-isotropic carbon fiber reinforced plastic laminates using a hydraulically driven fatigue tester and found that the surface temperature of the test piece increased by 80 °C at a frequency of 100 Hz. Xiao et al. [25] found that increasing the test frequency causes the temperature of AS4/PEEK specimens to rise significantly, which greatly reduces the fatigue strength. Holmes et al. [26] studied carbon fiber reinforced silicon carbide composites (C/SiC) and found that the test piece experienced a significant temperature increase when the test frequency changed from 1 to 85 Hz. They determined that this was due to the cyclic loading causing a mutual friction between fibers and between the fibers and the substrates. Staehler et al. [27] studied the high cycle fatigue behavior of C/SiC and proposed that the increase in the surface temperature of the test piece is related to the loading frequency and the cyclic stress applied. Backe et al. [16] used an ultrasonic device to study the very high cycle fatigue of carbon fiber resin composites using an intermittent loading while observing the test specimen with an infrared camera. They determined that the temperature increase in the test specimen at two locations was over 20 °C, whereas it increased rapidly over 45 ° C (Tmax = 45 °C ≈ 50% Tg) after delamination.

An ultrasonic fatigue test generally uses compressed cold air to cool the test piece. Silvain et al. [14] used compressed air to cool carbon fiber composite materials and effectively confine the surface temperature below 40 °C whereas it can increase above 90 °C in the absence of airflow. In this work, we used a thermal imaging of the CFRP specimens under cold air cooling. During loading, the highest temperature region of the test piece appeared above the two pivots yields of the specimen rather than where the test piece and the support point made contact. The high frequency friction inside the CFRP specimen caused a sharp increase in temperature that reached 106 °C in 7 s. The maximum temperature was 190 °C. Therefore, the use of compressed air cooling during ultrasonic loading of CFRP cannot guarantee a normal test.

The ultrasonic intermittent loading method is also used to control the temperature increase of the test piece and prevent it from becoming too high. Specifically, a control unit suspends the test for a given period of time after each ultrasonic excitation period. The intermittent time can be set manually. To prevent the temperature of CFRP specimens from increasing, Backe et al. [28] used compressed cold air to intermittently load CFRP specimens at a load frequency of 965 Hz. They applied an intermittent load for 1 second and stopped the test for 3 seconds. After the first load, the maximum temperature of the specimen was 42 °C, and the temperature dropped within 3 seconds after the load was stopped. However, after the second load was completed, the temperature rapidly increased to 60.5 °C. During the subsequent load, the maximum temperature of the test piece remained between 60 and 70 °C. When the loading cycle was N = 7.85 × 10^6^, the maximum temperature of the test was 78 °C. The temperature continued to increase during the subsequent loading sequence. When the loading cycle was N = 1 × 10^7^, the maximum temperature of the test piece exceeded 100 °C. Subsequently, the specimens were ablated by the high temperature and the loading frequency and the static loading dropped significantly. Therefore, the intermittent load method is also not suitable for CFRP ultrasound loading. When the loading cycle was N = 1.8 × 10^8^, the highest temperature was recorded between the fulcrum and the indenter and increased as the experiment progressed. When the cycle was N = 2 × 10^8^, the maximum temperature of the test piece was 68.3 °C and the highest temperature point appeared on the specimen. Then, when the temperature of the test piece increased, the static force and the frequency of the load decreased rapidly. The observations indicated that the test piece suffered fatigue failure. The cooling of the liquid nitrogen is needed to ensure the smooth conclusion of the fatigue test of the carbon fiber composites under ultrasonic loading.

**Figure 5:**
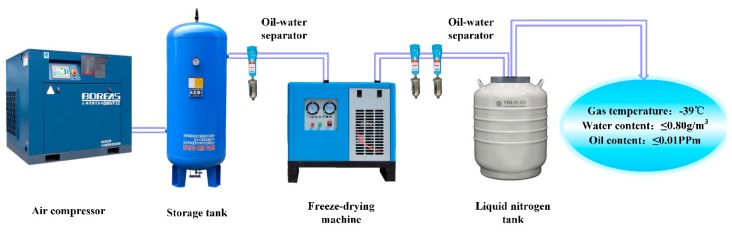
Liquid nitrogen cooling system.

Figure 5 shows the liquid nitrogen cooling system (Self-innovate, Xi’ an, China). The system uses the principle that liquid nitrogen vaporization can absorb a lot of heat to cool the compressed air. During the test, a metal tube is inserted into a liquid nitrogen tank. When the air flow passes through the tube, the liquid nitrogen absorbs heat and the air flow temperature drops rapidly. The air flow drives and covers the specimen to cool it. The minimum temperature of the air blown is −39 °C. During the test, the temperature of the test piece increased slowly. The maximum temperature of the contact point between the test piece and the fulcrum was kept below 40 °C and the loading frequency was maintained around 19730 Hz.

**Figure 6:**
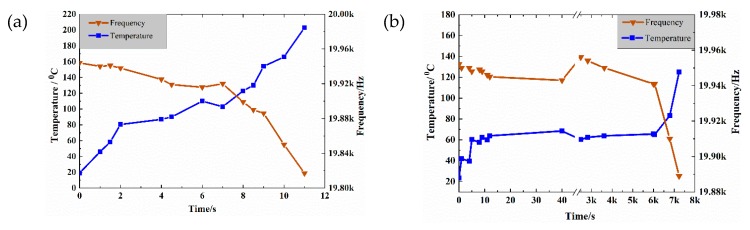

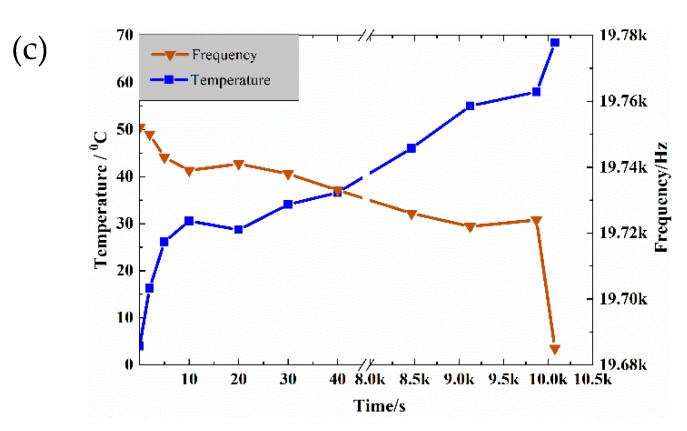
Evolution of the temperature (blue) and the frequency (red) when using (**a**) compressed cold air, (**b**) intermittent loading, and (**c**) liquid nitrogen cooling.

Figure 6 shows the changes in the temperature and the resonance frequency of the CFRP specimens under three loading and cooling modes. The three modes have very different cooling effects on the test specimen, but the compressed air is the worst. The temperature of the CFRP specimens can only be effectively controlled by using liquid nitrogen to guarantee the integrity of the CFRP specimens. When the temperature increases, the resonance frequency of the specimens decreases until the CFRP specimen is damaged or the resonance frequency cannot meet the test conditions of the system and the test is stopped.

## 3. Results and Discussion

### 3.1. S-N Curve

**Figure 7:**
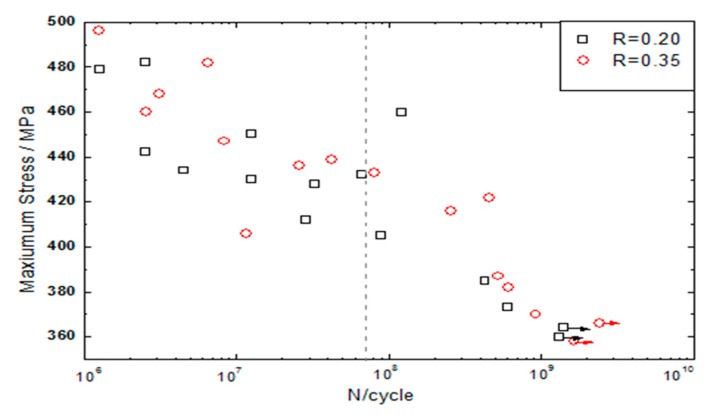
S–N curve for the CFRP specimen.

After a suitable cooling method was determined, the CFRP specimens were ultrasonically loaded and a stress ratio R of 0.2 and 0.35 was used. During the loading of the specimen, the test was stopped if the resonance frequency decreased by more than 10 Hz/s, the cycle number was >10^9^, or the temperature of the specimen surface was >90 °C. Figure 7 shows the corresponding fatigue S–N curve.

Figure 7 shows that both S–N curves decrease sharply when the cycle number is below 10^6^. When the cycle number is between 10^7^ and 10^8^, a horizontal plateau similar to the traditional fatigue limit appears. It indicates that the cycle number increases but the fatigue strength does not change. When the number of cycles is greater than 10^8^, the curve presents a second inflection and the fatigue strength continues to decrease. The S–N curve has a step shape because there is no fatigue limit similar to traditional definition of metal materials. Therefore, it is very risky to use a fatigue strength under 10^7^ as the strength design condition of CFRP very high cycle service members. The traditional fatigue limits of the CFRP specimens at R = 0.35 and 0.2 are 436 MPa and 428 MPa, respectively. Since the fatigue life of the material is lower for R = 0.2 than for R = 0.35, the ultrasonic dynamic load has a greater effect on the fatigue life of the specimen in the three point bending fatigue test carried out.

### 3.2. Characteristics of the VHCF

#### 3.2.1. Damage Morphology of the CFRP

**Figure 8:**
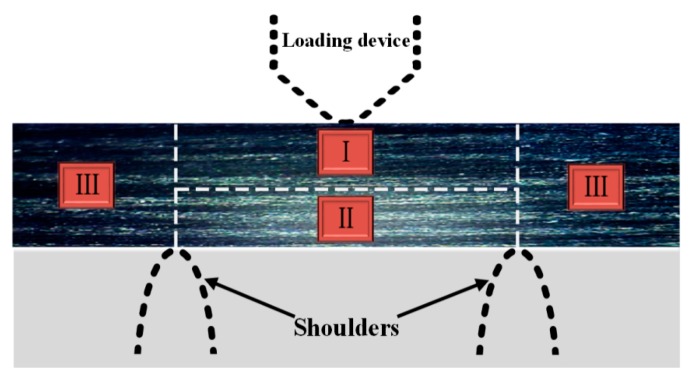
Division of the CFRP specimen.

The CFRP specimen is divided into regions I, II, and III, as shown in Figure 8. Region III is outside the two pivots as the free vibration end. Region I and II are bounded by the neutral surface of the specimen, so the material in region II is always subjected to tensile stress. With the change of stress ratio R, there are changes in tensile and compressive stress in region I.

**Figure 9:**
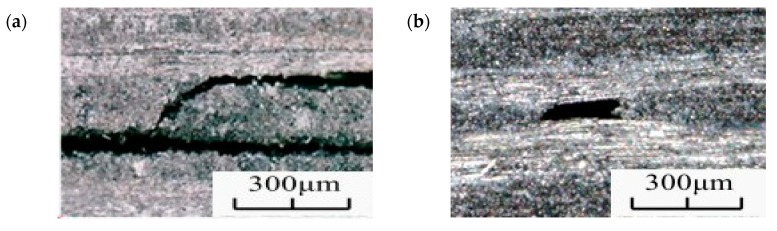
Fracture morphology of the CFRP specimen in (**a**) the HCF and (**b**) the VHCF regime.

In this work, the high cycle and the very high cycle fatigue tests of CFRP were performed by changing the stress (σ_u_, σ_m_) on the specimen and the morphology of the specimens was obtained by microscopic observation, as shown in Figure 9. The damage morphology has different characteristics. When comparing the high cycle and the very high cycle fatigues, the main fatigue failure forms in the high cycle fatigue specimens are layered and transverse cracks appear. The main failure forms of the very high cycle fatigue specimens are pitting corrosion.

**Figure 10:**
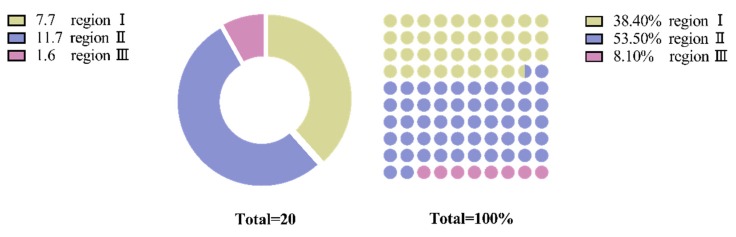
Statistics of the location of the pitting damage.

The location of the pitting corrosion was calculated from the microscopic observation of 20 specimens. Figure 10 shows that the pitting corrosion for the very high cycle fatigue specimens is mainly concentrated in regions I and II and that the proportions were not very different. Therefore, the pitting corrosion in the high cycle fatigue specimens is mainly caused by the vibration of the reciprocating micro-motion extrusion that causes damages to the weak part of the substrate. However, due to the low applied load, the damage cannot continue to expand and multiple corrosion pits are formed on the surface.

**Figure 11:**
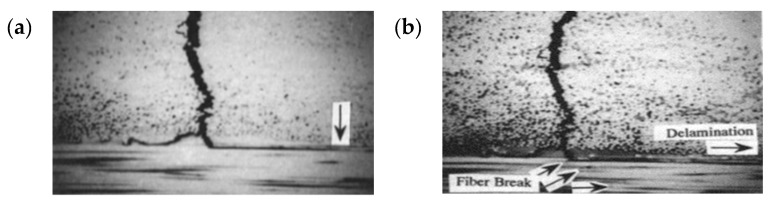
Transverse crack propagation in the LCF regime [29]: (**a**) transverse crack and stratification and (**b**) fiber fracture.

The low cycle fatigue (LCF) failure of CFRP starts from the transverse crack [22,23,24]. After the transverse crack expands, the stress concentration at the crack tip leads to delamination. In the case of serious damages, the fiber will break, as shown in Figure 11.

Because of the high loading stress, the low cycle fatigue failure is caused by micro-defects on the specimen activated before the vibration starts. With the cyclic loading, the large tensile stress promotes micro-defects to gradually expands laterally. After the intersection becomes a major crack and when the transverse cracks propagate to the layer interface, the transverse cracks are prevented due to the action of the fiber bundle. At this time, the stress concentration at the tip of the fatigue crack causes delamination.

When comparing the fracture morphology in the low cycle, the high cycle, and the very high cycle regimes, the main damage in the CFRP specimens with low cycle fatigue occurs in the form of transverse cracks and delamination. The main fatigue failure of the high cycle fatigue specimens mainly consists of delamination and transverse cracks whereas the main failure morphology of the very high cycle fatigue specimens is pitting corrosion.

#### 3.2.2. Damage Evolution in the CFRP Specimens

**Figure 12:**
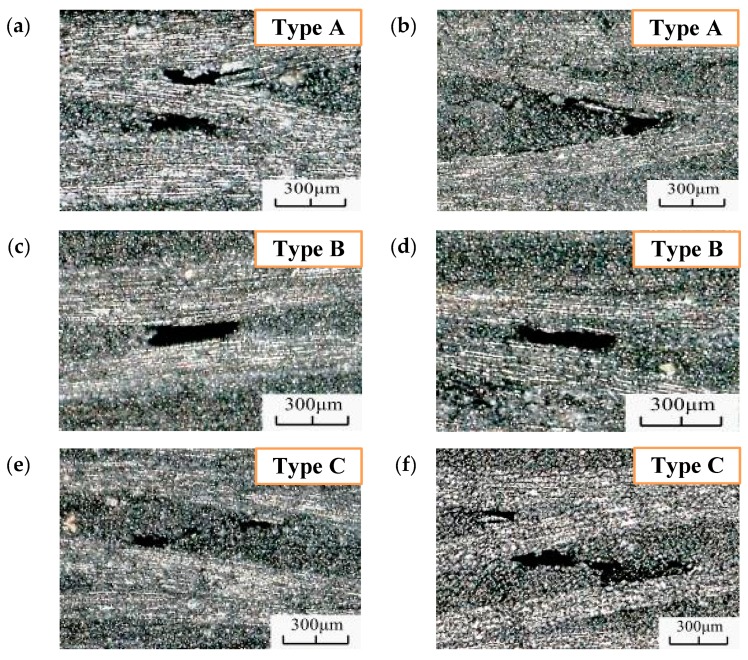
Fatigue characterization in the VHCF regime for (**a**) N = 5.52 × 10^8^, R = 0.35, (**b**) N = 1.26 × 10^8^, R = 0.35, (**c**) N = 3.46 × 10^8^, R = 0.35, (**d**) N = 8.64 × 10^8^, R = 0.2, (**e**) N = 2.23 × 10^9^, R = 0.2, and (**f**) N = 3.55 × 10^9^, R = 0.2.

The fatigue morphology of the specimens was evaluated via optical microscopy, as shown in Figure 12. The figure shows several pitting corrosions on the different specimens. Three different failure morphologies were observed for the VHCF: matrix damage at the intersection of the fiber bundles (Type A), matrix cavity near the parallel section of the fiber bundles (Type B), and matrix penetration (Type C). The fiber bundle intersection deformation structure resulted from the intersection of the fiber bundles, and owing to this intersection, the material bonding force was reduced, resulting in easy destruction of the fibers; the void morphology of the parallel segments of the fiber bundles, the distance between the upper and lower fiber bundles was small, and the influence on the matrix increased. Consequently, the base body was relatively weak and prone to damage; the relatively large base through-topography lies on the base with a large fiber bundle spacing.

**Figure 13:**
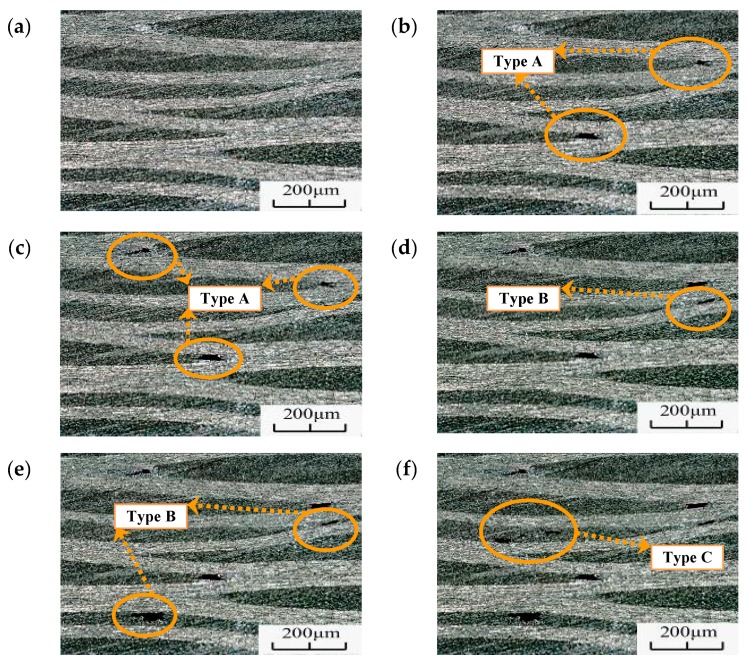
Fatigue failure morphology after different weeks for the same field of view (R = 0.35, σ_max_ = 368 MPa) for (**a**) N = 0, (**b**) N = 7.6 × 10^7^, (**c**) N = 1.45 × 10^8^, (**d**) N = 6.23 × 10^8^, (**e**) N = 8.86 × 10^8^, and (**f**) N = 2.17 × 10^9^.

To understand the formation characteristics and mechanism of the failure morphology, the microscope was placed on the side of the specimen, which was then monitored during the test (shooting interval: 3 s). The generated fatigue morphology is shown in Figure 13. As the figure shows, when N = 1.76 × 10^7^, type A morphology appeared under this field of view. This morphology appeared first, indicating that the interlaminar adhesion was relatively weak at the fiber intersection sites, and fatigue failure morphology was generated relatively easily under cyclic load. Type B morphology was generated at N = 6.23 × 10^8^, as shown in Figure 13d. The fiber bundles at this location were close to each other, resulting in a weaker binding force than those at other locations and easy fatigue failure. When N = 8.86 × 10^9^, type C morphology appeared in the field of view. At this point, the loading frequency of the specimen would drop rapidly and a measurement instability would occur. The surface temperature of the sample would increase gradually with loading and, hence, the test was considered complete.

These observations indicated that the occurrence of the damage morphology differs with the conditions and the severity of this damage increases with increasing load cycle. The fatigue damage on the order morphology is presented. The concrete matrix for the fiber bundle parallel segment became empty before the segment matrix nearly parallel to the fiber bundle, which became empty before the matrix. Therefore, $\frac{1}{t_{A}}>\frac{1}{t_{B}}>\frac{1}{t_{C}}$.

**Figure 14:**
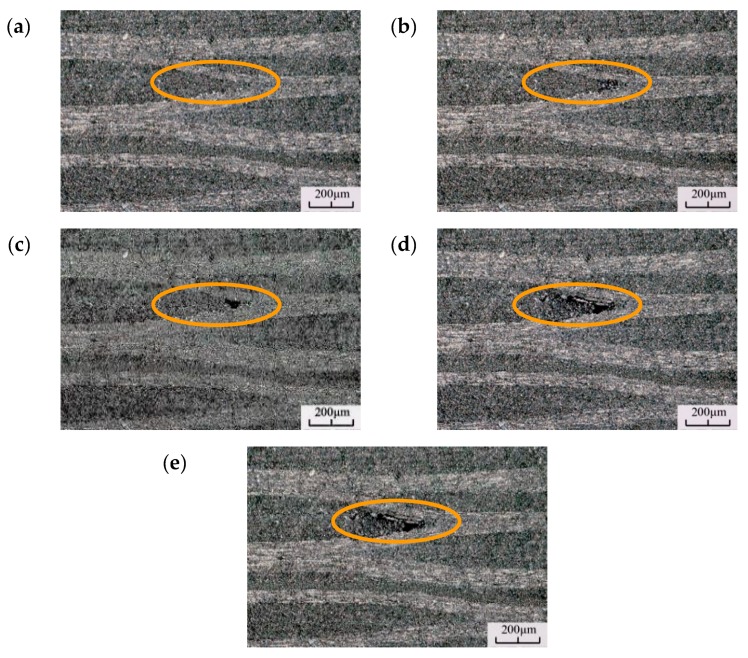
Evolution and expansion of the pitting morphology for (**a**) N = 6.0 × 10^7^, f = 20,130 Hz, (**b**) N = 8.9 × 10^7^, f = 20,126 Hz, (**c**) N = 9.7 × 10^7^, f = 20,118 Hz, (**d**) N = 2.5 × 10^8^, f = 20,105 Hz, and (**e**) N = 3.8 × 10^8^, f = 20,086 Hz.

To understand the occurrence of pitting morphology, the discontinuity time of the portable microscope was reduced to 1 s. When the specimen cycle was 6 × 10^7^, recording was started to capture the initiation and expansion of a single pitting failure, as shown in Figure 14.

Figure 14a is the micrograph of the specimen without pitting damage. The fiber bundles have multiple intersections at this location, which is prone to matrix destruction at the intersection of the fiber bundles. Figure 14b shows that a dark area was generated at the fiber crossing and the matrix was darker than the surrounding, but no obvious pitting occurred. Figure 14c shows the gradual expansion and deepening of the dark area at the fiber intersection. The color becomes darker there, but no pitting morphology is observed and the area remains dark. The fatigue failure continues to evolve. Figure 14d–f shows the pitting morphology at different cycles. The pitting size does not increase significantly when the cycle number increases, which means that the damage expands to a certain size then stops.

### 3.3. Mechanical Properties

**Figure 15:**
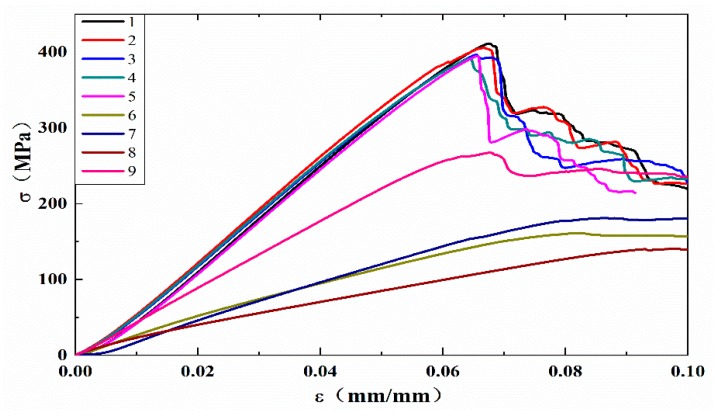
The σ-ε curve for the static loading.

The influence of the HCF on the material was evaluated from the fatigue failure during the static three point bending test. Four samples were subjected to very high cycle fatigue failure (Figure 15). The untested CFRP samples are plotted as curves 1–5. The fatigue life corresponding to curves 6, 7, and 8 were N = 5.6 × 10^8^, N = 1.75 × 10^8^, and N = 8.28 × 10^8^ cycles, respectively.

**Figure 16:**
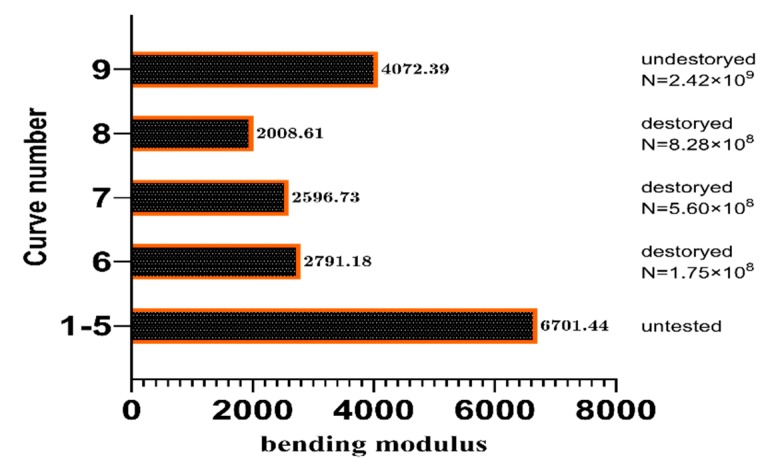
Flexural modulus of the CFRP specimens tested.

The calculated flexural modulus for curves 6–9 is listed in Figure 16. Compared to curves 1–5 and 6–8, the average flexural modulus of the test specimen (2465.51 MPa) is 63.2% lower than that of the untested CFRP specimens (6701.44 MPa). The VHCF has a significant effect on the mechanical properties of the CFRP. This is because of the pitting corrosion that occurs during VHCF and reduces the bonding force between the layers of the material. During loading, the pits are the sites of the damage initiation and the time required for the extended response decreases.

Curves 6 and 8 show a 6.97% and 22.6% decrease compared with curve 7, respectively. This indicates that the flexural modulus of the CFRP specimen decreases when the fatigue life increases. However, the decrease was low, which suggests that the bending of the material under the same fatigue failure mode yields approximately the same modulus. Combined with the results of the HCF tests, it suggests that the fatigue mode of the material plays a major role in the deterioration of the mechanical properties.

Curve 9 (N = 2.42 × 10^9^) corresponds to the static loading curve of the specimen subjected to more than 10^9^ loading cycles, where the test was stopped in accordance with the criteria determined earlier. The specimen only showed a slight VHCF morphology. For example, the flexural modulus (4072.39 MPa) is 9.2% lower than that of the untested specimen, illustrating again the effect of VHCF on the mechanical properties of the material.

### 3.4. Evolution Threshold

The fatigue morphology of the CFRP evolved during the transition from HCF to VHCF. Therefore, establishing a threshold for HCF to VHCF can provide a basis for studying and preventing the failure of the CFRP.

Given the anisotropy of the composites, the traditional stress intensity factor K cannot be used. Therefore, the strain energy release rate G that has been widely used to study the interlaminar fracture of composites was selected. The strain energy release rate G can be expressed as [30,31]:

(6) $$G=\frac{18P\delta a^{2}}{b(L_{0}{}^{3}+12a^{3})}.$$

where *P*: loading force; *δ*: deflection corresponding to *P*; *a*: crack length, which represents the length of type A pitting corrosion; *b*: specimen width; *L*_0_: test span.

(7) $$G_{\max}=\frac{18P_{\max}\delta_{\max}a^{2}}{b(L_{0}{}^{3}+12a^{3})}.$$

The fatigue test provides loading at different stress ratios, therefore:

(8) $$R=\frac{P_{\max}}{P_{\min}}=\frac{\delta_{\max}}{\delta_{\min}}.$$

The fatigue crack expansion threshold ∆G can be expressed as:

(9) $$∆G=G_{\max}-G_{\min}.$$

Substituting Equations (7) and (8) into Equation (9) yields:

(10) $$∆G_{th}=(1-R^{2})G_{(\max)th}.$$

The rate of expansion of the interlayer crack and the maximum strain energy release rate $G_{\max}$ are numerically related, which indicates that $G_{\max}$ is proportional to the crack growth rate [14]. Therefore, when testing the VHCF, the specimen susceptibility to VHCF should decrease with $∆G_{th}$.

## 4. Conclusions

In this paper, tests on CFRP were conducted using an ultrasonic three point bending fatigue test device to investigate its behavior during very high cycle fatigue. The following conclusions were drawn:

(1) A liquid nitrogen cooling device was designed and its efficiency was compared to that of compressed cold air cooling and intermittent loading.
(2) According to the S-N curve of CFRP material, the evolution process and competition mechanism of CFRP from high cycle fatigue to very high cycle fatigue are revealed, and the evolution threshold is established.
(3) The comparison of the flexural modulus of the specimens before and after the test indicated that very high cycle fatigue significantly reduced the flexural modulus of the specimen. Consequently, the influence of VHCF on the material properties was manifested as a significant decrease in the bending modulus of the specimen.
(4) In situ microscopic observations showed that a very high cycle fatigue failure morphology was obtained in the CFRP. The different fatigue failure morphologies were generated in a different order.

## Figures and Tables

**Table 1:** Properties of the composite material used.

*E*_1_/GPa	*E*_2_/GPa	*E*_3_/GPa	*υ*_12_	*υ*_13_	*υ*_23_	*G*_1_/GPa	*G*_2_/GPa	*G*_3_/GPa
144.70	9.65	9.65	0.30	0.30	0.45	5.2	5.2	3.4
